# Selection of a Gentamicin-Resistant Variant Following Polyhexamethylene Biguanide (PHMB) Exposure in *Escherichia coli* Biofilms

**DOI:** 10.3390/antibiotics10050553

**Published:** 2021-05-10

**Authors:** Clémence Cuzin, Paméla Houée, Pierrick Lucas, Yannick Blanchard, Christophe Soumet, Arnaud Bridier

**Affiliations:** 1 Antibiotics, Biocides, Residues and Resistance Unit, Fougères Laboratory, French Agency for Food, Environmental and Occupational Health & Safety (ANSES), 35300 Fougères, France; clemence.cuzin@gmail.com (C.C.); pamela.houee@anses.fr (P.H.); christophe.soumet@anses.fr (C.S.); 2 Viral Genetics and Biosecurity Unit, Ploufragan-Plouzané-Niort Laboratory, French Agency for Food, Environmental and Occupational Health & Safety (ANSES), 22440 Ploufragan, France; pierrick.lucas@anses.fr (P.L.); Yannick.blanchard@anses.fr (Y.B.)

**Keywords:** biocide, antibiotic resistance, cross-resistance, aminoglycoside, adaptation, biofilm, pyruvate cycle

## Abstract

Antibiotic resistance is one of the most important issues facing modern medicine. Some biocides have demonstrated the potential of selecting resistance to antibiotics in bacteria, but data are still very scarce and it is important to better identify the molecules concerned and the underlying mechanisms. This study aimed to assess the potential of polyhexamethylene biguanide (PHMB), a widely used biocide in a variety of sectors, to select antibiotic resistance in *Escherichia coli* grown in biofilms. Biofilms were grown on inox coupons and then exposed daily to sublethal concentrations of PHMB over 10 days. Antibiotic-resistant variants were then isolated and characterized phenotypically and genotypically to identify the mechanisms of resistance. Repeated exposure to PHMB led to the selection of an *E. coli* variant (Ec04m1) with stable resistance to gentamycin (8-fold increase in minimum inhibitory concentration (MIC) compared to the parental strain. This was also associated with a significant decrease in the growth rate in the variant. Sequencing and comparison of the parental strain and Ec04m1 whole genomes revealed a nonsense mutation in the *aceE* gene in the variant. This gene encodes the pyruvate dehydrogenase E1 component of the pyruvate dehydrogenase (PDH) complex, which catalyzes the conversion of pyruvate to acetyl-CoA and CO_2_. A growth experiment in the presence of acetate confirmed the role of this mutation in a decreased susceptibility to both PHMB and gentamicin (GEN) in the variant. This work highlights the potential of PHMB to select resistance to antibiotics in bacteria, and that enzymes of central metabolic pathways should be considered as a potential target in adaptation strategies, leading to cross-resistance toward biocides and antibiotics in bacteria.

## 1. Introduction

In recent years, the dramatic rise of antimicrobial resistance (AMR) in bacteria has emerged as one of the most challenging concerns for global health. Identifying the drivers of AMR, especially anthropogenic ones, is thus of prime importance to better control the emergence and spread of resistant bacteria [1,2]. In industries, medical areas, public spaces or at home, biocides are broadly used to avoid the dissemination of pathogenic bacteria and guarantee the microbiological quality of equipment, production surfaces and products. However, an increasing number of studies reported the potential of different biocides to impact antibiotic susceptibility in various bacterial species [3,4,5]. Indeed, some mechanisms responsible for a lower susceptibility in adapted bacteria following a biocide exposure could be also involved in antibiotic resistance, as for instance an upregulated non-specific efflux mediated by multidrug pumps, one of the most recurrently described [6,7,8]. The adaptation of bacterial populations to biocides particularly occurs in the presence of sublethal concentrations of biocides which select the most tolerant or resistant subpopulations. Different phenomena are at the origin of these sublethal concentrations in industries or at a hospital, for instance as a result of misuse during biocide application or the presence of interfering organic substances [9,10]. In addition, the development of biofilms on surfaces has an effect on biocide concentrations actually experienced by bacteria. Indeed, biofilms are surface-associated bacterial communities embedded in an extracellular matrix, and often exhibit specific functions compared to planktonic cells such as better resistance to disinfectants [11]. The three-dimensional structure of biofilm is known to be able to hinder biocide penetration to the deeper layers, resulting in biocide concentration gradients across the biofilm depth [12,13]. Internal layers can thus be in the presence of lower biocide concentrations that enable bacteria to survive, and trigger an adaptive response to such sub-inhibitory biocide concentrations.

Polyhexamethylene biguanide (PHMB) has been a commonly used biocide for some considerable time, employed in a wide variety of sectors such as disinfectant and antiseptic formulations for wound therapy, in cosmetics, in poultry production to prevent *Salmonella,* or in swimming pool cleanser [14,15]. Despite this broad utilization, data about its potential to select resistance toward other antimicrobials as antibiotics are very scarce and require novel dedicated studies. In this context, we investigated the effects of repeated exposure to PHMB on the antibiotic susceptibility profiles of bacteria in *E. coli* biofilm.

## 2. Results

### 2.1. Colony Morphotype Identification Following PHMB Exposure of Ec04 biofilms

**Figure 1:**
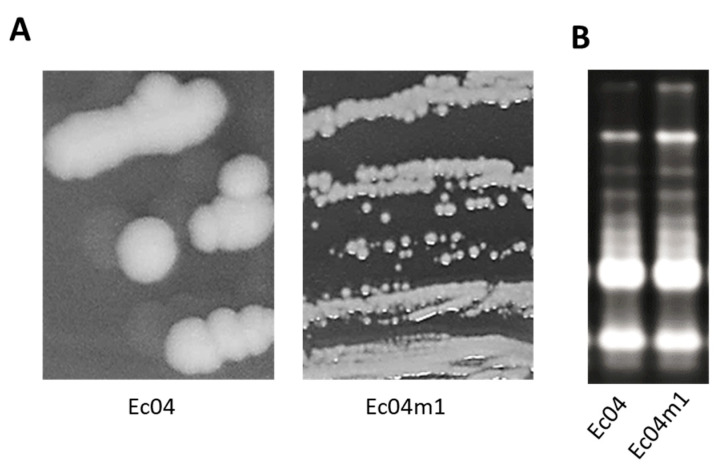
(**A**) Colony morphotypes obtained after plating of Ec04 biofilm exposed to PHMB over 9 days. Ec04m1 corresponds to a small colony variant. (**B**) ERIC-PCR profiles of Ec04 and Ec04m1.

Biofilms of the Ec04 strain on stainless steel coupons were exposed daily over 9 days to 1.5625 mg L^−1^ of PHMB, corresponding to sublethal concentrations. Cells were then recovered and plated on trypticase soy agar (TSA) after dilutions. Two different morphotypes were detected, a small colony variant morphotype (Ec04m1) and the parental colony morphotype (Figure 1A). Both morphotypes displayed identical Enterobacterial Repetitive Intergenic Consensus Polymerase Chain Reaction (ERIC-PCR) profiles, suggesting Ec04m1 is not a contamination (Figure 1B).

### 2.2. PHMB and Antibiotic Minimal Inhibitory Concentrations (MIC) Determination for Ec04 Derivatives

Antibiotic and PHMB MIC were determined for the morphotype Ec04m1 in comparison with the parental Ec04. An increase in MIC was observed for Ec04m1 for PHMB (2-fold increase) and two antibiotics, gentamicin (GEN, 8-fold increase) and trimethoprim (TMP, 4-fold increase) (Table 1). These increases led to MIC values of 8 mg L^−1^ and 4 mg L^−1^ for GEN and TMP, respectively, above the ECOFF defined by EUCAST for both antibiotics. A 2-fold decrease in AMP MIC was conversely observed in Ec04m1.

To assess the stability of this loss of susceptibility toward PHMB, GEN and TMP in the Ec04m1 variant, 10 daily successive subcultures were carried out in a medium without PHMB, and MICs were determined for subcultures at days 2, 5, 7 and 10. The results confirmed the stability of the PHMB, GEN and TMP MIC increase in Ec04m1, as no evolution of MICs was observed after 10 days (Table 1).

### 2.3. Growth Rate Parameters in Presence of PHMB and GEN

**Figure 2:**
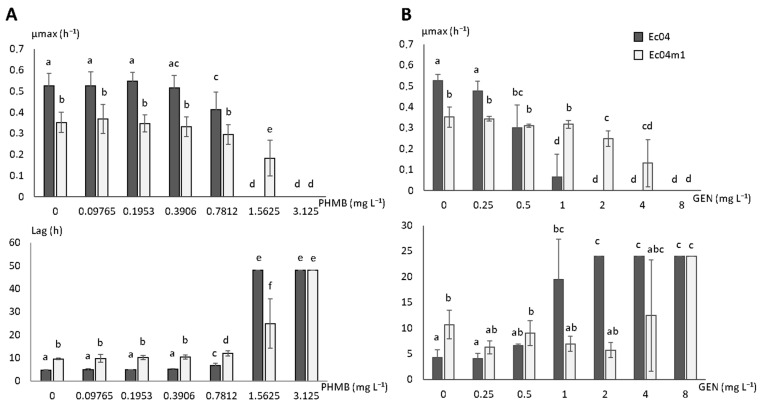
Maximum growth rate (µmax) and lag time (lag) for parental strain Ec04 and Ec04m1 variant in the presence of (**A**) PHMB concentrations from 0 to 3.125 mg L^−1^, or (**B**) GEN from 0 to 8 mg L^−1^. Different letters upon the SD bars indicate significant differences between µmax or lag mean values (T-test. *p* < 0.05).

The growth rates of Ec04m1 in gradual concentrations of PHMB or GEN were compared to those obtained from the Ec04 parental strain and are presented in Figure 2. The time to reach the maximal growth rate was also calculated (lag time).

The Ec04m1 variant exhibited a significant decrease in growth rate of 33%, and a 2-fold increase in lag time compared to the parental strain Ec04 in 1/10 TSB medium. A significant decrease (*p* < 0.05) in the µmax value was observed for Ec04 strain from a PHMB concentration of 0.7812 mg L^−1^ (Figure 2A). No significant decrease in the µmax value was observed for Ec04m1 at this PHMB concentration (*p* = 0.0968). Lag times were nevertheless significantly increased for both strains at this concentration. At 1.5625 mg L^−1^ of PHMB, a concentration corresponding to the MIC value determined for Ec04, and no growth was detected for this strain, whereas the Ec04m1 variant was able to grow with a µmax equal to 0.183 h^−1^. At 3.125 mg L^−1^ of PHMB, growth was inhibited for both strains. These results underlined the ability of the variant Ec04 to survive in higher PHMB concentrations compared to the parental Ec04 strain.

Comparable results were obtained with GEN (Figure 2B). Indeed, the µmax and lag time were deeply affected by GEN from a concentration of 1 mg L^−1^ (*p* < 0.05) in the Ec04 strain, whereas no effect was observed on growth parameters at this concentration for the Ec04m1 variant. While the growth of Ec04 strain is fully inhibited at 2 mg L^−1^, the µmax obtained for Ec04m1 variant gradually decreased from 2 to 8 mg L^−1^, confirming its lower susceptibility to this aminoglycoside antibiotic.

### 2.4. Genomic Characterization of Ec04m1 Variant

**Figure 3:**
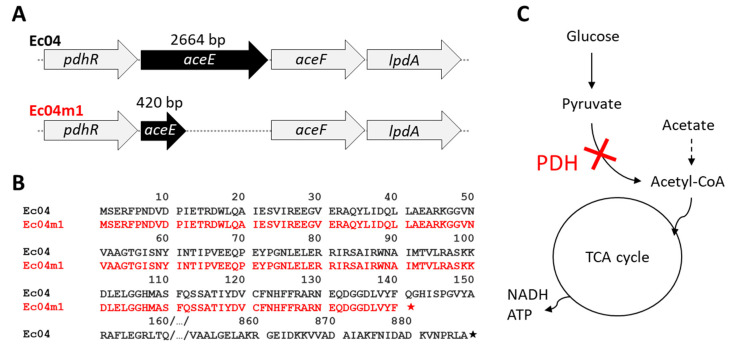
Detection of a nonsense mutation in the Ec04m1 variant in *aceE* gene encoding the pyruvate dehydrogenase E1 component of the pyruvate dehydrogenase (PDH) complex that catalyzes the conversion of pyruvate to acetyl-CoA and CO_2_. The mutation (C > T at position 421) results in a premature stop codon at the location of glutamine, leading to a truncated *aceE* protein (140aa). (**A**) Schematic representation of the *aceE* gene and its genetic environment (*pdhR-aceEF-lpdA* operon) in Ec04 and Ec04m1 strains. (**B**) Protein sequence of *aceE* in Ec04 (887aa) and its alignment with the truncated protein in Ec04m1 (140aa); stars symbolize stop codons. (**C**) Schematic representation of the central metabolic pathway altered by the mutation in the Ec04m1 variant.

The genomes of strains Ec04 (biosample SAMN16976434) and Ec04m1 (SAMN16976489) were sequenced and compared to investigate the genetic origin of the Ec04m1 variant phenotype. Only one SNP with a minimum frequency >80% was identified in the Ec04m1 variant compared to Ec04, leading to the substitution of cytosine by thymine at position 421 in the *aceE* gene (Figure 3A). This mutation results in a stop codon at the location of glutamine, leading to a truncated AceE protein in the Ec04m1 variant (140aa versus 887 in the parental Ec04 strain) (Figure 3B). AceE is the pyruvate dehydrogenase (PDH) E1 component and forms with AceF and LpdA, the PDH enzyme complex normally catalyzing the conversion of pyruvate in acetyl CoA under the regulation of a pyruvate-sensing PdhR regulator. The mutation detected in Ec04m1 thus impairs acetyl-CoA production from pyruvate, and alters a central pathway critical for the tricarboxylic acid (TCA) cycle (Figure 3C).

### 2.5. Effect of Acetate on Growth Rate in the Presence of GEN and PHMB

**Figure 4:**
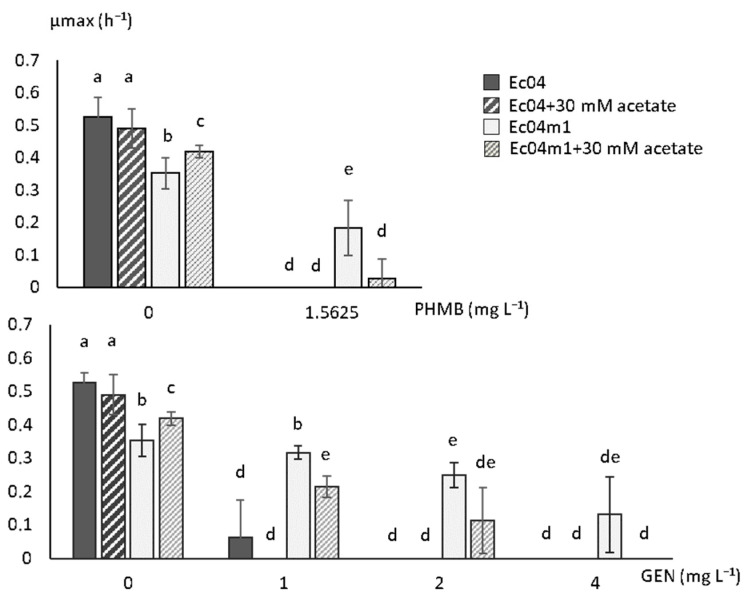
Effect of acetate medium supplementation (30 mM) on maximal growth rate (µmax) for parental strain Ec04 and Ec04m1 variant in the presence of PHMB or GEN. Different letters upon the SD bars indicate significant differences between µmax or lag mean values (T-test. *p* < 0.05).

The effect of acetate addition in the growth medium on the growth rates of the Ec04m1 variant in the presence of PHMB or GEN was assessed, since alternative pathways for the synthesis of acetyl-CoA from acetate exist in *E.coli*. In the absence of PHMB and GEN, the presence of acetate led to a slight but significant increase in growth rate in the Ec04m1 variant (*p* < 0.05) although it remains below that measured for Ec04 (Figure 4). At a PHMB concentration of 1.5625 mg L^−1^, Ec04m1 growth was almost totally inhibited in the presence of 30 mM acetate, as in the Ec04 parental strain, while the variant was able to grow in the absence of acetate under identical conditions as previously shown. Similar observations were made in the presence of GEN. As shown in Figure 4, at an antibiotic concentration of 1 and 2 mg L^−1^, the presence of acetate led to a significant decrease in the Ec04m1 growth rate, although remaining significantly higher than that observed for Ec04. The presence of 30 mM acetate resulted in the inhibition of Ec04m1 growth at a GEN concentration of 4 mg L^−1^, while Ec04m1 was able to grow at this antibiotic concentration in the absence of acetate. Overall, the supplementation of acetate in the growth medium led to a partial restoration of the susceptibility of the Ec04m1 strain to both PHMB and GEN.

## 3. Discussion

Large quantities of biocides are used every day to prevent the spread of pathogenic microorganisms in a wide variety of sectors. An increasing quantity of evidence emphasizes a connection between biocide exposure and the modification of susceptibility toward antibiotics in bacteria [3,4]. A better understanding of the potential of various biocides to select resistance toward antibiotics, along with the associated mechanisms, is therefore required in the perspective to combat antibiotic resistance emergence. In this study, we highlighted the potential of 1.5625 mg L^−1^ PHMB exposure to select resistance to antibiotics, especially GEN, in *E. coli*. This sublethal concentration is approximately 100 times lower than the end-use concentration, for instance when disinfecting hard surfaces (0.16 g L^−1^, [16]). Previous studies have shown a decrease in susceptibilities toward antibiotics in various bacterial species after exposure to sublethal concentrations of chlorhexidine, another member of the biguanide molecule family [17,18]. Interestingly, Henly et al. [19] reported that PHMB exposure induced resistance to trimethoprim-sulfamethoxazole in the CFT073 *E. coli* strain, and resistance to GEN in the EC26 strain, which is consistent with the results obtained in the present work. Here, the Ec04m1 variant also exhibited a 2-fold increase in PHMB MIC after repeated exposure. This suggests the mechanisms leading to this lower susceptibility due to adaptation to PHMB could be shared with those playing a role in antibiotic resistance. Polymeric biguanides, such as PHMB, first act on bacteria through the interaction with cations associated with the cell envelope, causing membrane destabilization and LPS reorganization [20]. This cellular uptake mechanism is shared with polycationic agents, such as aminoglycosides like GEN, and therefore modifications of membrane permeability in variant Ec04m1, altering cellular uptake, could explain both decreases in susceptibility to PHMB and GEN as proposed previously [19].

WGS revealed the presence of a unique mutation in Ec04m1 compared to the Ec04 parental strain in the *aceE* gene, which encodes the pyruvate dehydrogenase E1 component. The mutation resulted in a truncated protein in the variant, impairing the conversion of pyruvate in Acetyl-CoA, a central pathway in carbon metabolism in *E. coli*. Consistently with the significant decrease in growth rate observed in the Ec04m1 variant in comparison with the Ec04 parental strain (Figure 2), it was previously shown that *aceE* deletion greatly affected growth rate and biosynthetic capacity in *E. coli* [21,22]. Globally, the depletion of enzymes of the pyruvate cycle, such as pyruvate dehydrogenase, has been considered to shut down the TCA cycle, and thus to profoundly affect energy production and regulation in *E. coli*. Interestingly, Schutte et al. [21] showed that the pyruvate dehydrogenase complex is a central component in the antimicrobial activity mediated by the chemokine CXCL10 in *E. coli*. Changes in carbon metabolism activity are also known to affect bacterial susceptibility toward antibiotics including aminoglycosides in *E. coli* [23,24]. Recently, it was also shown that inactivation of central carbon metabolism enzymes can also participate in antibiotic resistance in other Gram-negative species such as *Stenotrophomonas maltophilia* [25]. In line with this, stimulation of the central metabolism using various metabolites enabled researchers to potentiate aminoglycoside efficacy in *E. coli* or *Pseudomonas* spp. through the stimulation of the proton motive force (PMF) favoring the uptake of antibiotics across the bacterial membrane [23,26,27]. As reported by Chindera et al. [14], PHMB also enters the bacterial cell through an energy-dependent uptake process, since authors showed that bacteria cultivated at 4 °C displayed reduced PHMB uptake compared to cells held at 37 °C. The lower susceptibility of the Ec04m1 variant selected upon PHMB exposure could thus be related to the reduced uptake of antimicrobial molecules due to the altered pyruvate cycle and reduced metabolic activity. Moreover, the deletion of *aceE* greatly alters fatty acid biosynthesis, which is linked to the central carbon cycle through the use of acetyl-CoA [22]. This biosynthesis pathway is crucial for lipid synthesis in the cell envelope, emphasizing a relevant connection between the alteration of the central carbon biosynthesis pathway in the Ec04m1 variant and modification of the membrane permeability, which could therefore play a role in decreased susceptibility to both PHMB and GEN, as previously suggested.

To confirm the role of the mutation in *aceE* in the decreased susceptibility to GEN and PHMB, observed in the Ec04m1 variant, growth rate measurements were performed in the presence of 30 mM acetate. Acetate addition has been shown to suppress the effects of *aceE* deletion in *E. coli,* allowing the synthesis of acetyl-CoA by an alternative pathway (Figure 3C) [28]. Our results revealed that acetate growth medium supplementation did indeed result in a significant increase in the growth rate of the Ec04m1 variant and resulted in a partial restoration of its susceptibility to both PHMB and GEN (Figure 4). This observation thus confirmed the involvement of the pyruvate cycle alteration in the adaptation to biocides after repeated exposure, and the development of GEN resistance. While this central metabolism alteration penalizes the Ec04m1 variant in the absence of biocides or antibiotics by decreasing its growth rate, the fact that it was favored at low PHMB concentrations (1.5625 mg L^−1^) emphasizes its potential ability to survive in environments where residual concentrations of PHMB are present. PHMB, in addition to being used in a wide variety of applications, is a chemically stable molecule, especially in water, and thus can persist for a long time in the environment at these low concentrations [29]. Moreover, the presence of interfering substances or biofilms can reduce biocide concentrations that reach bacteria [12]. Such phenomena are likely to create favorable micro-environmental conditions for the survival and dissemination of the resistant variant, thus representing a risk for public health.

## 4. Materials and Methods

### 4.1. Bacterial Strain and Growth Conditions

The *E. coli* strain used in this study (called hereafter Ec04) was initially isolated from a pig caecum in a slaughterhouse (Brittany, France). Bacterial stock cultures were kept at −80 °C in a cryoprotective solution (0.5% tryptone, 0.3% beef extract, 15% glycerol). Prior to each experiment, frozen cells were firstly sub-cultured on a trypticase soy agar (TSA) plate over 24 h at 37 °C, and a colony was then transferred to tryptone soya broth (TSB) at 37 °C overnight.

Biofilms were grown on sterile 10 × 20 × 1 mm^3^ stainless steel coupons by inoculating wells of a 6-well microtiter plate with 4 mL of overnight bacterial suspension adjusted in 1/10 TSB to 10^3^ CFU mL^−1^. The 6-well microplate was then incubated at 20 °C for 72 h to enable the formation of the biofilm on the coupons at a cell density of approximately 10^7^ CFU/cm^2^.

### 4.2. Biocide Susceptibility Testing

PHMB MIC were determined using a microdilution broth method adapted from that of Schug et al. [30]. Briefly, each well of a 6-well microtiter plate was inoculated with 2 mL of biocide at the desired concentration, and 2 mL of a planktonic suspension was obtained after 72 h growth at 20 °C in 1/10 TSB and then adjusted to 10^7^ CFU mL^−1^. Plates were then incubated at 20 °C for 24 h (also checked at 48 h) and MIC was determined as the lowest concentration of biocide that prevents bacterial growth. MIC values were determined through the reading of turbidity and confirmed by drop-plating on TSA plates to confirm the reading through colony growth examination. All the determinations of MIC were repeated at least twice.

### 4.3. Antibiotic Susceptibility Testing

Antibiotic susceptibility tests were performed using a standard microdilution method (EUVSEC, Sensititre^®^, TREK Diagnostic Systems Ltd., Thermo Fisher Scientific, East Grinstead, UK), using a panel of 14 antimicrobial substances according to manufacturer instructions. The strains were interpreted as resistant to antibiotics according to the epidemiological resistance cut-off value (ECOFF) determined by EUCAST (European Committee on Antimicrobial Susceptibility Testing, http://mic.eucast.org, accessed on 14 April 2021). *E. coli* ATCC 25,922 was used as quality control. All the determinations of MIC were repeated at least twice, and thrice if values were not similar.

### 4.4. Adaptation Experiments to PHMB and Identification of Colony Morphology Variants

Biofilms were repeatedly exposed to a sublethal concentration of PHMB at 1.5625 mg L^−1^ corresponding to the MIC obtained for planktonic suspension of the Ec04 strain. Concretely, biofilm coupons were daily transferred into new wells of 6-well microtiter plates filled with fresh 1/10 TSB medium containing PHMB at 1.5625 mg L^−1^, over 9 days, and then biofilm coupons were transferred in a neutralizing agent (REF + composition). Coupons were then ultra-sonicated and vortexed to recover biofilm cells. Serial dilutions were then performed and cells were plated on TSA. The different morphotypes were then checked on the TSA plate, and each colony with a distinct morphology was isolated and stocked in a cryoprotective medium at –80 °C before further experiments.

ERIC-PCR was used to compare fingerprint patterns obtained with morphotypes and the Ec04 parental strain to ensure that the different colony morphotypes did indeed correspond to Ec04 derivatives and not to a contamination event. To further this aim, DNA was extracted using an InstaGene kit (Bio-rad, Marnes-la-Coquette, France) and amplified using a LightCycler^®^ 480 thermocycler (Roche Diagnostics, Meylan, France) with primers ERIC1-R (ATGTAAGCTCCTGGGGATTCAC) and ERIC2 (AAGTAAGTGACTGGGGTGAGCG) [31] and GoTaq Flexi polymerase (Promega, Charbonnières-les-bains, France) as follows: 95 °C for 2 min for initial melting; 30 cycles at 95 °C for 1 min, 54 °C for 1 min, 72 °C for 4 min; final extension at 72 °C for 8 min followed by incubation at 4 °C. PCR products were then checked on 1% agarose gel and migrated over 90 min at 110 V before being revealed using a GelRED stain (Biotium, Brumath, France).

MIC for biocides and antibiotics were determined as described earlier, to evaluate changes in antimicrobial resistance profiles, due to repeated PHMB exposure, for confirmed morphotype variants. In the case of MIC, it increased compared to WT. Daily subcultures over 10 days in fresh TSB without PHMB were performed, and MICs were determined again on the cells after days 2, 5, 7 and 10, to assess the stability of the potential modification of antibiotic susceptibility as observed.

### 4.5. Growth Parameter Measurements

The growth parameters of bacterial strains were determined by automatically measuring the OD of cultures at 620 nm in a FLUOstar Optima microplate reader (BMG Labtech, Champigny sur Marne, France) in the presence or absence of PHMB or GEN. Briefly, 4 mL of TSB were inoculated from cryotubes and incubated at 37 °C for 24 h. Then, 300 µL from this suspension was transferred in 4 mL TSB and incubated for 6–7 h at 37 °C. The bacterial suspension was adjusted to 10^3^ CFU mL^−1^ and used to inoculate the 96 wells of a microtiter plate (Greiner) containing 1/10 TSB (with or without 30 mM acetate) and a range of concentrations of PHMB (0–3.125 mg L^−1^) or GEN (0–8 mg L^−1^). OD_620nm_ was then automatically measured every 30 min over 48 h at 37 °C by the FLUOstar reader. Maximum growth rate (µ_max_) values and lag time (λ) were extracted from OD curves using MARS data analysis software (version 2.10, BMG Labtech, Champigny sur Marne, France). Experiments were performed at least three times independently of duplicates.

### 4.6. WGS and Mutation Detection

Genomic DNA was extracted from the pellet of a 5 mL exponential culture of strains Ec04 or Ec04m1, grown in TSB centrifuged at 8000 g for 5 min using the Nucleospin tissue kit (Macherey-Nagel, Duren, Germany) according to manufacturer instructions. The quantity and quality of the DNA were checked using a BioSpec-Nano (Shimadzu, Marne la Vallée, France) spectrophotometer. Whole-genome sequencing was performed with the NextSeq 500 (Illumina). Reads were assembled using the Shovill pipeline (v0.9.0, https://github.com/tseemann/shovill (accessed on 30 April 202).). This pipeline used Trimmomatic (v0.38) and SPAdes (v3.13.0). All contigs with a length shorter than 200 nucleotides and a kmer coverage lower than 2 are filtered. Genomes were annotated with Prokka [32] (v1.13.3), then a BWA-MEM [33] (v0.7.8) alignment was performed using the Ec04 genome as a reference against all reads cleaned by Trimmomatic (ILLUMINACLIP: oligos.fasta: 2:30:5:1: true; LEADING: 3; TRAILING: 3; MAXINFO: 40:0.2; MINLEN: 36). Variant calling was performed using the bwa alignment file with VarScan [34] (version 2.4; parameters: min-coverage, 8; min-reads2, 2; min-avg-qual, 15; min-var-freq, 0.2; *p* value, 0.05; strand-filter disabled; variants 1).

### 4.7. Sequence Accession Numbers

Whole-genome sequence assemblies have been deposited in NCBI in bio-project PRJNA681999, with bio-sample numbers SAMN16976434 for Ec04 and SAMN16976489 for Ec04m1.

## 5. Conclusions

This study revealed the potential of PHMB exposure to select resistance toward antibiotics in *E. coli* through the alteration of central carbon flow. These observations emphasize the ability of bacterial populations to adapt to various antimicrobial stresses by reshaping their central metabolism, which might constitute an important target to be considered when understanding cross-resistance emergence between biocides and antibiotics.

## Figures and Tables

**Table 1:** Minimal inhibitory concentrations (MIC, mg L^−1^) for 14 antibiotics and PHMB in Ec04 and derivatives.

Strain	AMP	AZI	FOT	TAZ	CHL	CIP	COL	GEN	MER	NAL	SMX	TET	TIG	TMP	PHMB
Ec04	8	8	0.25	0.5	8	0.015	1	1	0.03	4	16	64	0.25	1	1.5625
Ec04m1	4	8	0.25	0.5	8	0.015	1	8	0.03	4	16	64	0.5	4	3.125
Ec04m1_D2	4	8	0.25	0.5	8	0.03	1	8	0.03	4	32	64	0.5	4	3.125
Ec04m1_D5	2	8	0.25	0.5	8	0.03	1	8	0.03	4	32	64	0.25	4	3.125
Ec04m1_D7	4	8	0.25	0.5	8	0.03	1	8	0.03	4	32	64	0.25	4	3.125
Ec04m1_D10	4	8	0.25	0.5	8	0.03	1	8	0.03	4	32	64	0.25	4	3.125

Ec04m1_D2, Ec04m1_D5, Ec04m1_D7 and Ec04m1_D10 respectively correspond to Ec04m1 after 2, 5, 7 or 10 days of subculture in TSB without PHMB. AMP: ampicillin, AZI: azithromycin, FOT: cefotaxime, CHL: chloramphenicol, CIP: ciprofloxacin, COL: colistin, GEN: gentamicin, MER: meropenem, NAL: nalidixic acid, SMX: sulfamethoxazole, TAZ: ceftazidime, TET: tetracycline, TIG: tigecycline, TMP: trimethoprim.

## Data Availability

All data were available in the manuscript. Sequencing data were deposited in NCBI public database under the bio-project PRJNA681999.
